# Editorial

**Published:** 1884-05

**Authors:** 


### EDITORIAL.

The annual meeting of the State Dental Society of the State of Michigan was held in Detroit on the 26th, 27th and 28th of March. The occasion was one of interest and profit. The disposition to have the members conform to the laws of the society, and especially to the code of ethics was clearly manifested here, as it has recently been in some other societies. Three or four members had, during the past year, swerved somewhat from a full compliance with the code. After a full and free discussion of the matter the offenders saw their course in a different light, and expressed regrets for the past, and promised a change in conduct for the future, all of which was very gratifying to those present.

There is a disposition on the part of many of our State societies to insist upon conformity on the part of members to the code of ethics; and this, undoubtedly, is the correct course. If one may, with impunity, violate the code of ethics, another may, and, indeed, all may, and thus an end would come to all restriction, and the profession would sink to the low level of charlatanry.

Prof. A. W. Harlan, of Chicago, was present, and added much to the interest of the occasion by a clinical lecture on diseases and treatment of the gums, which was highly appreciated by all present. On this subject Dr. H. is certainly abreast with the most advanced views and practice.

The Board of Examiners, acting under the law regulating the practice of dentistry in the State, was in session. Several applicants for examination appeared before them. A large per cent. of them was found to be without proper qualifications, and were advised to further pursue their course of study. This is as it should be. In Michigan there is no excuse for this board authorizing incompetent men to practice dentistry. In, comparatively, the near future the dental profession in Michigan will occupy a much higher position than hitherto. This statement is made without any thought of comparing the profession of this State with that of any other; and with the full conviction that some of the best men in the profession reside in Michigan.

Long live the Michigan Dental Society.

